# Supplementary material for: Age mosaic of gut epithelial cells prevents aging
Source: Nat Commun. 2025 Jul 22;16:6734. doi: 10.1038/s41467-025-62043-y (PMC12284232; doi:10.1038/s41467-025-62043-y)
Supplement: Supplementary file 1 — Supplementary Information [file 41467_2025_62043_MOESM1_ESM.pdf]

Age mosaic of gut epithelial cells prevents aging

Peizhong Qin[1], Qi Wang[2], You Wu[1], Qiqi You[1], Mingyu Li[1], Zheng Guo[1,3*]

[1]Department of Medical Genetics, School of Basic Medicine, Institute for Brain Research, Tongji Medical College, Huazhong University of Science and Technology, Wuhan 430022, China

[2]Department of Urology, Union Hospital, Tongji Medical College, Huazhong University of Science and Technology, Wuhan 430022, China

[3]Cell Architecture Research Center, Huazhong University of Science and Technology, Wuhan, 430030, China

*Correspondence: guozheng@hust.edu.cn

Supplementary Figures:

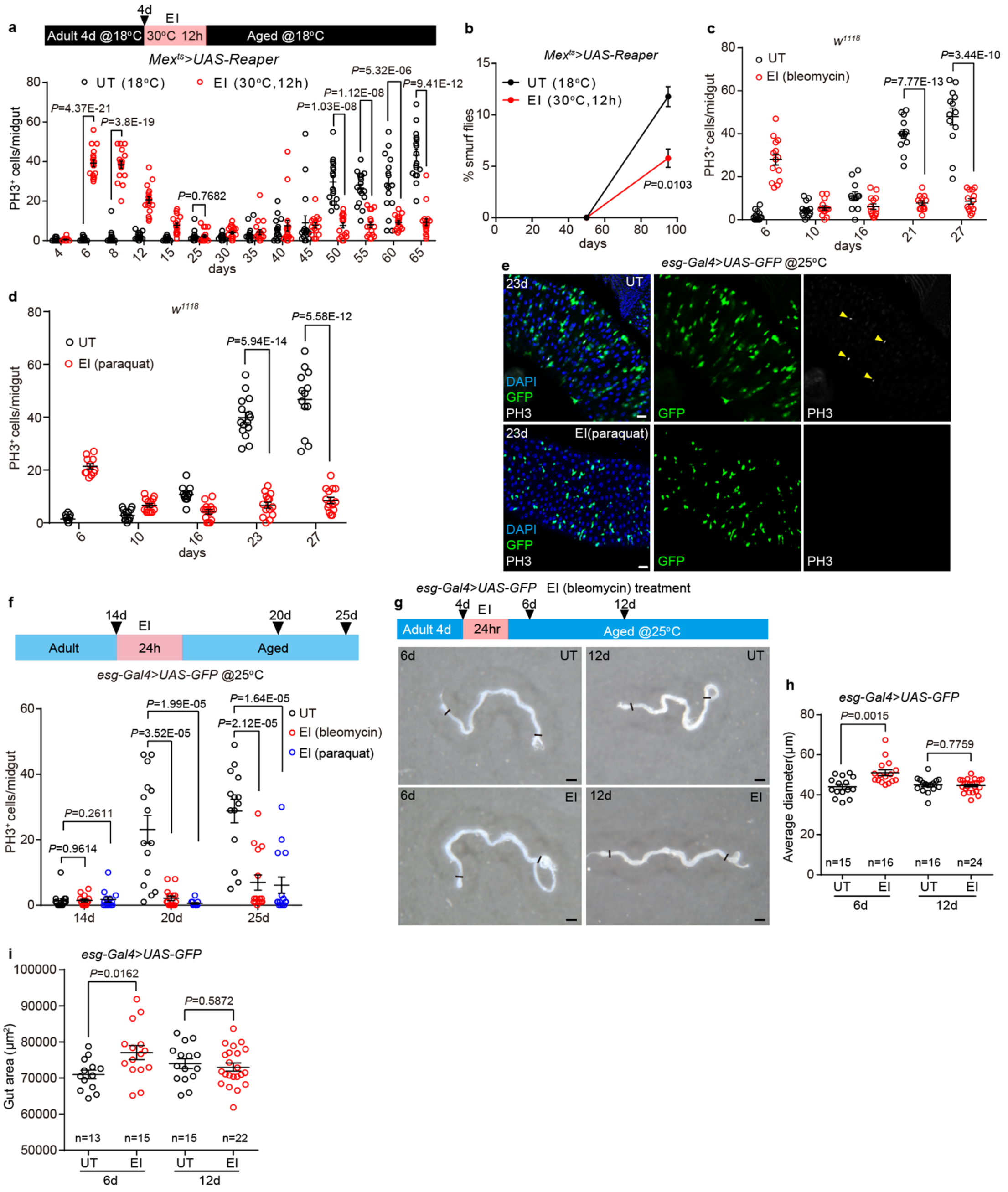

Supplementary Fig. 1 | Early injury alleviates midgut hyperplasia in aged flies.

a, Time course of the statistics of PH3^{+} mitotic ISCs per midgut in UT and EI flies. Flies were transferred to 30°C for 12h at 4d to induce a transient EC ablation (*Mex^{ts}>UAS-Reaper*). n, number of midguts, the exact n numbers are provided in Source Data file. **b,** Statistics of percentage of smurf flies in UT and EI group (*Mex^{ts}>UAS-Reaper*). n, number of flies. UT: n=60, 60. EI: n=55, 55. **c, d,** Time course of statistics of PH3^{+} cells per midgut in *W^{1118}* untreated (UT) and early injury (EI) flies. Early injury of bleomycin **c** or paraquat **d** treatment was performed on 4d mated adult female flies for 24h. n, number of midguts, the exact n numbers are provided in Source Data file. **e,** Representative images of posterior midguts stained with GFP and PH3 (white) in aged (23d) UT and EI (paraquat) *esg-Gal4>GFP* flies. Yellow arrowheads indicate the positions of PH3^{+} cells. **f,** Upper, schematic illustration of the method that EI was induced at 14d, before the onset of midgut hyperplasia. Midguts were dissected at the indicated time 14d, 20d and 25d.

Lower, statistics of PH3⁺ cells per midgut at 14d, 20d, 25d. n, number of midguts. n (from left to right) =15, 14, 13, 15, 15, 14, 14, 15, 15. **g,** Representative image of the midgut of UT and EI flies at 6d and 12d. **h, i,** Quantification of the average diameter of the R4C-R5A region of the midgut and the midgut area in UT and EI flies. n, number of midguts. Data are mean ± SEM. Significance was determined using two-tailed unpaired *t* test. Scale bars, 20 μm (**e**), 200 μm (**g**). Source data are provided as a Source Data file.

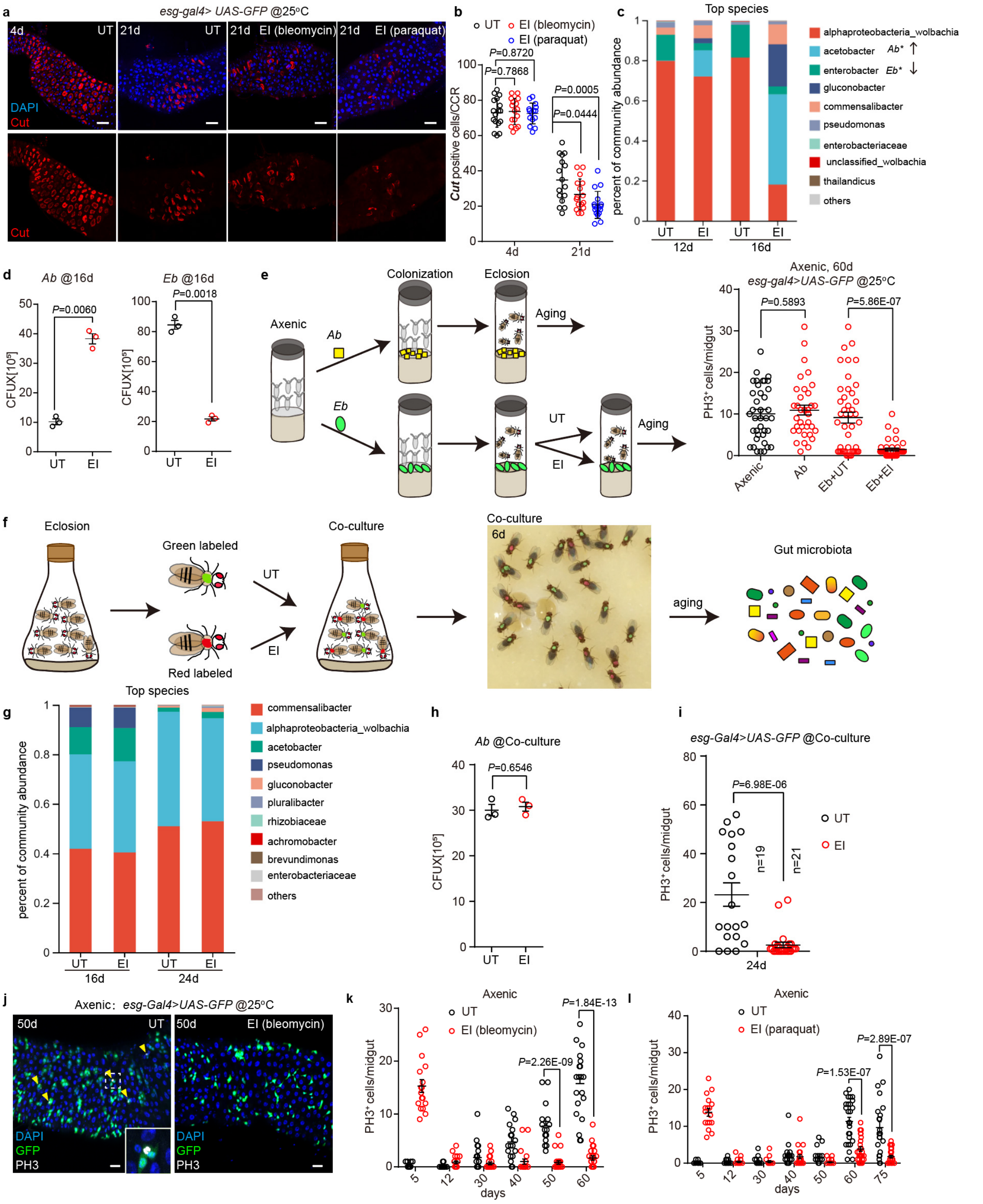

Supplementary Fig. 2 | EI induced hyperplasia alleviation is not due to compartmentalization or microbiota change.

a, Representative image of the copper cell region of UT and EI flies at 4d and 21d. Cut staining (red) labels copper cells. **b,** Statistics of the number of Cut⁺ cells in the CCR of UT and EI files at 4d and 21d. n, numbers of midguts. n (4d, from left to right) =17, 16, 15; n (21d, from left to right) =16, 16, 17. **c,** Commensal composition of 12d and 16d UT

and EI (bleomycin-fed for 24h at 4d) midguts. Histogram charts show percentages of top bacterial species determined by 16S rRNA sequencing. Each represents the average of four samples (4x20 midguts). **d,** Statistics of the number of *Ab* and *Eb* colony forming units (CFU) by culturing 16d UT and EI (bleomycin-fed for 24h at 4d) midgut commensals on selection plates. n=3 replicates. **e,** Left; Cartoon showing the procedure of colonization of flies with a single bacterium (*Ab* or *Eb*). Right; Statistics of PH3$^+$ cells per midgut in 60d flies reared under the indicated conditions. n=38 (UT), 35 (Ab), 44 (Eb+UT), 37 (Eb+EI). **f,** The cartoon shows the procedure of co-culturing green thorax-tagged UT flies with red thorax-tagged EI flies in the same cage to form the same commensal colonization in both UT and EI flies. **g,** Commensal composition of co-cultured 16d and 24d UT and EI (bleomycin-fed for 24h at 4d) midguts. Each represents the average of three samples (3x20 midguts). **h,** Statistics of the number of *Ab* CFU by culturing 24d co-cultured UT and EI (bleomycin-fed for 24h at 4d) midgut commensals on selection plates. n=3 replicates. **i,** Statistics of PH3$^+$ cells in 24d co-cultured UT and EI midgut. n=19 (UT), 21 (EI). **j,** Representative images of 50d UT and EI (bleomycin-fed for 24h at 4d) posterior midgut reared on axenic food. Insets represent the close-up views of PH3$^+$ staining in the selected region. Yellow arrowheads indicate the positions of PH3$^+$ cells. **k, l,** Time course of the statistics of PH3$^+$ cells in UT and EI midguts reared on axenic food. Bleomycin-fed for 24h at 4d (**k**) and paraquat-fed for 24h at 4d (**l**). n, numbers of midguts, the exact n numbers are provided in Source Data file. Data are mean ± SEM. Significance was determined using two-tailed unpaired *t* test. Scale bars, 50 μm **a** or 20 μm **j**. Source data are provided as a Source Data file.

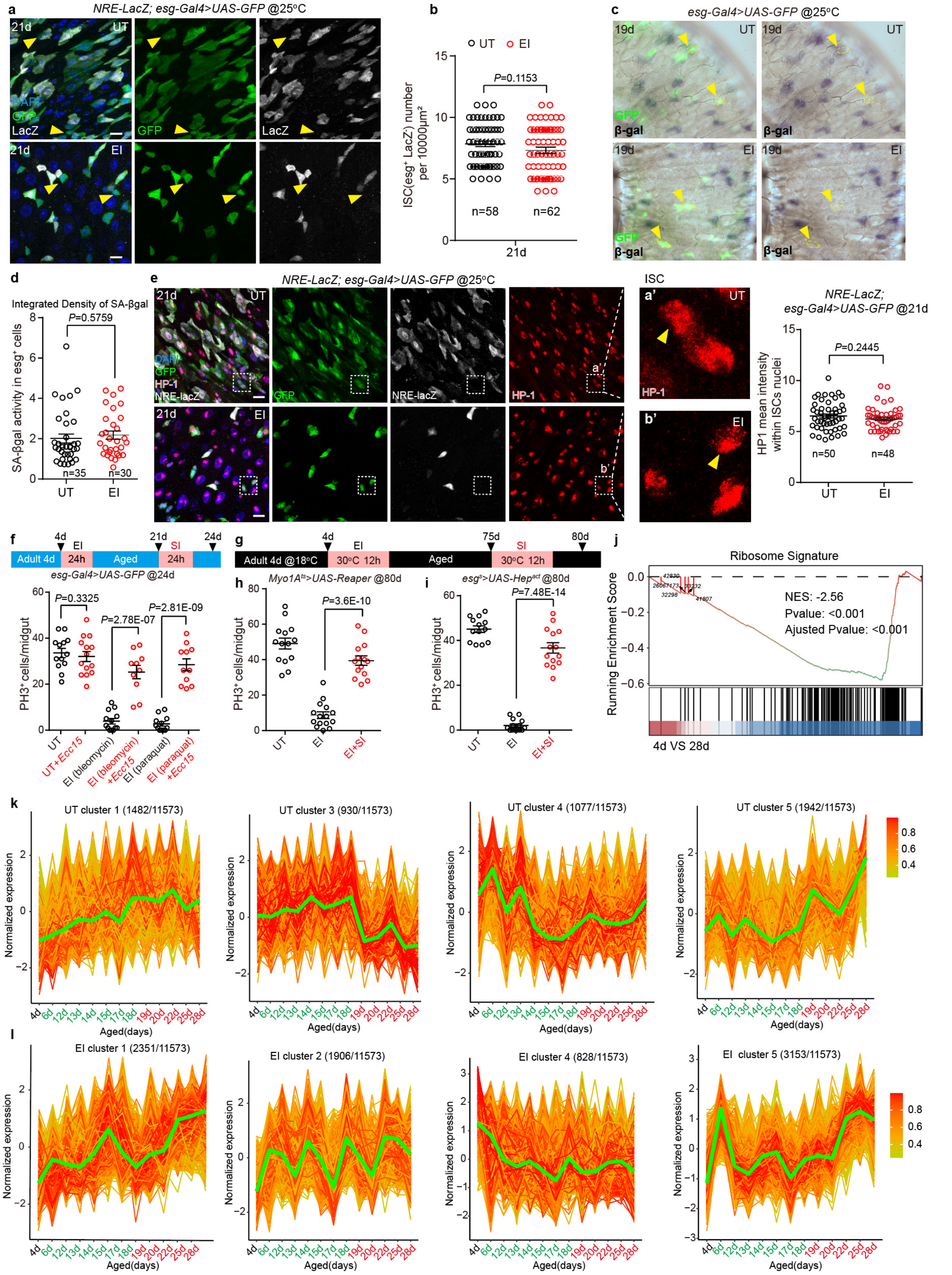

Supplementary Fig. 3 | EI alleviates abrupt transcriptome changes without affecting ISC number and causing ISC senescence

a, b, Representative images **a** and statistics **b** of the ISC number in 21d UT and EI (bleomycin-fed for 24h at 4d) posterior midgut. ISCs were determined as *esg*$^+$ *NRE-LacZ*$^-$ cells. Yellow arrowheads indicate the positions of the ISCs. n, number of regions. **c, d,** Representative images **c** and statistics **d** of the intensity of senescence-associated β-galactosidase (SA-βgal) activity in 19d UT and EI posterior midgut. Yellow arrowheads indicate the positions of the ISC/EB progenitor cells (GFP). n, number of ISC/EB. n=35 (UT), 30 (EI). **e,** Representative images and statistics of the

intensity of the senescence-associated marker HP1 staining in ISCs (GFP$^+$ LacZ$^-$ cells) of 21d UT and EI posterior midgut. **a'** and **b'** show higher magnification of HP1 staining in ISCs. Yellow arrowheads indicate the positions of ISCs. n, number of ISCs. n=50 (UT), 48 (EI). **f,** Upper, the design of Second Injury (SI) experiments to test the hypothesis of EI induced ISC senescence. The SI was performed by *Ecc15* oral infection for 24h at 21d. Lower, statistics of PH3$^+$ cells in 24d UT and EI midgut. n, numbers of midguts. n (from left to right) =13, 13, 13,10, 12,11. **g,** The design of SI by transferring *Myo1A^{ts}>UAS-Reaper* or *esg^{ts}>UAS-Hep^{act}* flies to 30°C for 12h to induce EC death or JNK activation at 75d to test the hypothesis of EI induced ISC senescence. Midguts were dissected and stained with PH3 at 80d. **h, i**, Statistics of PH3$^+$ cells of UT, EI and EI+SI midguts at 80d. *Myo1A^{ts}>UAS-Reaper* induced the SI is **(h)** and *esg^{ts}>UAS-Hep^{act}* induced the SI is **(i)**. n, numbers of midguts. n (h: UT, EI, EI+SI) =14, 15, 14. n (i: UT, EI, EI+SI) =11, 14, 14. **j,** 4d vs. 28d midgut Gene set enrichment analysis (GSEA) plot of expression of ribosome signature identity genes. **k, l,** Temporal trend analyses of all genes in the UT and EI groups identified five clusters of gene trajectories that change with age, respectively. Except for UT cluster 2 and EI cluster 3 (Fig. 2e), the remaining 4 UT clusters (**k**) and 4 EI clusters (**l**) are shown here. The green lines represent the average trajectory for each cluster. Data are mean ± SEM. Significance was determined using two-tailed unpaired *t* test. Scale bars, 10 μm (**a, c, e**). Source data are provided as a Source Data file.

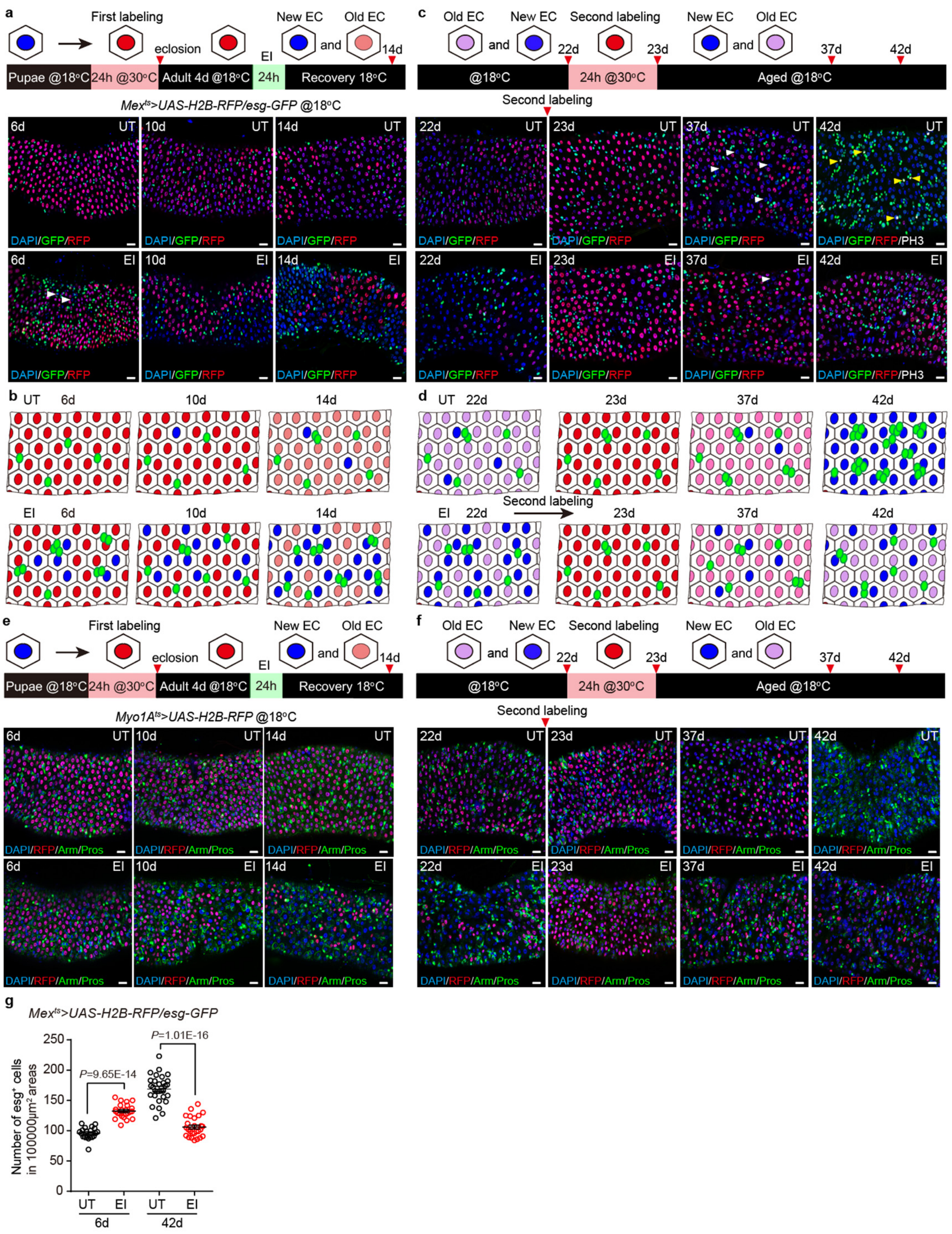

Supplementary Fig. 4 | The age mosaic induced by EI prevents the abrupt onset of aging hyperplasia

a, Upper, schematic illustration of the method to track the turnover of puparium labeled H2B-RFP$^+$ ECs in UT and EI (bleomycin-fed 24h at 4d) midguts. Lower, representative images of the tracing pattern of H2B-RFP$^+$ ECs and *esg*-GFP labeled ISC/EBs in UT and EI midguts at 6d, 10d and 14d. Midgut regions were selected from R4C/R5A. Newly generated ECs (white arrowheads) are RFP$^-$ and visualized by the large polyploid DAPI staining. **b,** Cartoon showing the distribution of puparium-labeled RFP$^+$ (red) ECs versus newly generated RFP$^-$ (blue) ECs and *esg*-GFP$^+$ (Green cells) ISC/EBs in the UT and EI midgut during aging. The red color, representing the amount of H2B-RFP in the EC nuclear, fades with age. Green cells are ISC-EB pairs. **c,** Upper, schematic illustration of the second H2B-RFP labeling and tracing of ECs. Lower, representative images of the tracing pattern of RFP$^+$ ECs and *esg*-GFP$^+$ ISC/EBs in UT and

EI midguts after 22d. White arrowheads indicate the newly generated RFP⁻ ECs, yellow arrowheads indicate PH3⁺ cells. **d,** Cartoon showing the abrupt collective turnover of RFP⁺ ECs from 37d to 42d, contrasting with the formation of the age mosaic of EI midgut. **e,** Upper, schematic illustration of the method to track the turnover of puparium labeled H2B-RFP⁺ ECs in UT and EI (bleomycin-fed 24h at 4d) midguts. Lower, representative images of the tracing pattern of H2B-RFP⁺ ECs and the ISC/EB marker Armadillo (Arm) and the EE marker Prospero (Pros) staining in UT and EI midguts at 6d, 10d and 14d. **f,** Second H2B-RFP labeling and tracing of ECs. White arrowheads indicate the newly generated RFP⁻ ECs. **g,** Quantification of the number of *esg*-GFP⁺ cells in midguts of UT and EI flies (**a** and **c**). n, numbers of regions. 6d: n=20 (UT), n=23 (EI); 42d: n=30 (UT), n=26 (EI). Data are mean ± SEM. Significance was determined using two-tailed unpaired t test. Scale bars, 20 μm.

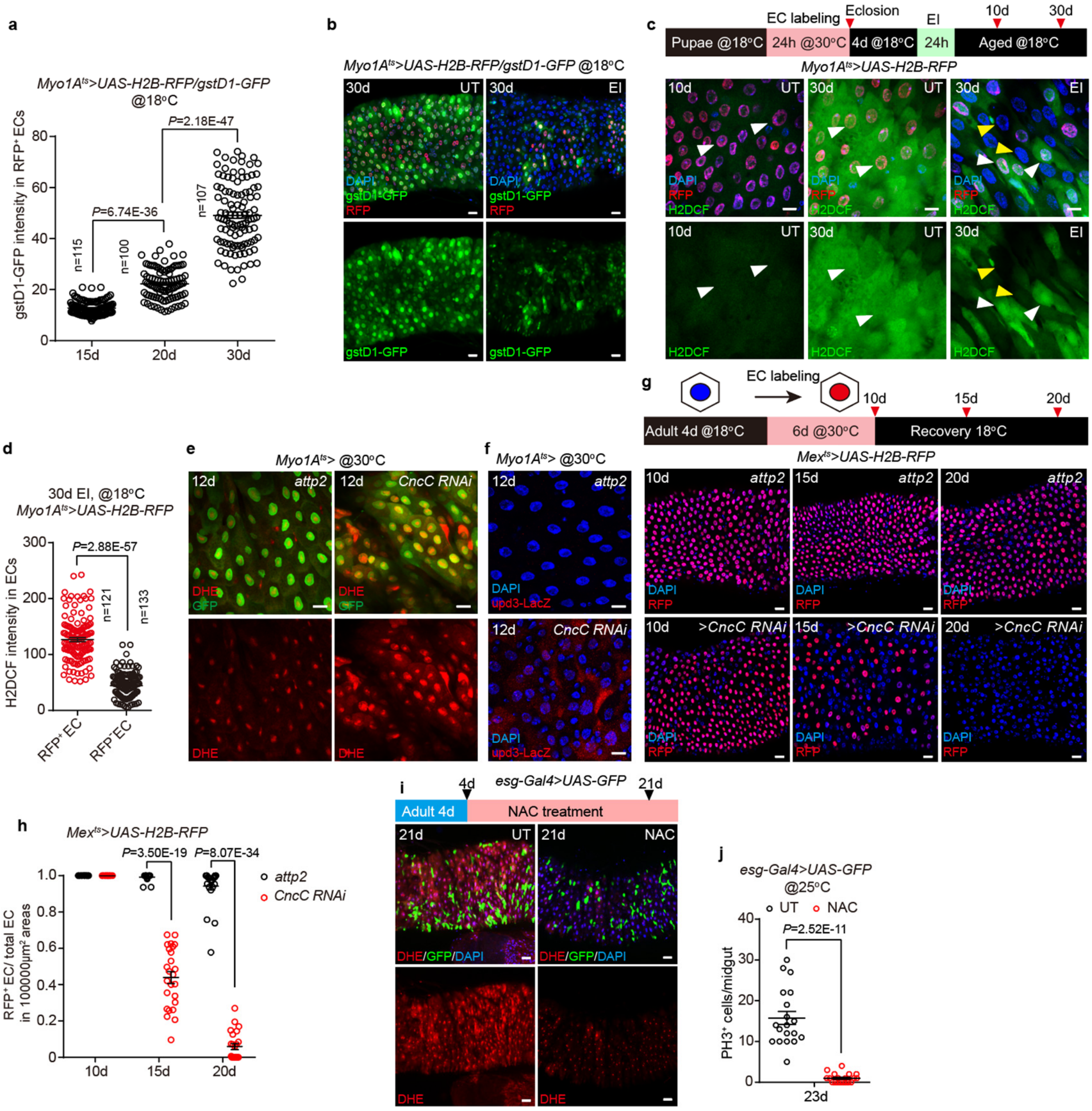

Supplementary Fig. 5 | Age-related accumulation of ROS in ECs

a, Statistics of *gstD1*-GFP intensity in RFP⁺ ECs of 15d, 20d and 30d flies during aging. n, numbers of ECs. **b,** Representative images of *gstD1*-GFP staining in 30d UT and EI (bleomycin-fed 24h at 4d) midgut. **c,** Upper: The schematic shows the method for measuring the level of ROS in vivo using H2DCF in the midgut with puparium H2B-RFP labeled ECs. The midgut was dissected at 10d and 30d. Lower: representative images of H2DCF staining in UT and EI midguts. White arrowheads point to puparium-labeled RFP⁺ ECs and yellow arrowheads point to new generated RFP⁻ ECs. **d,** Statistics of H2DCF intensity in RFP⁺ (old) ECs or RFP⁻ (new) ECs in EI midguts at 30d. n, numbers of ECs. **e,** Representative images of DHE staining in control (*attp2*) and *CncC* knockdown (*Myo1A^{ts}*>*CncC RNAi*) posterior

midgut. ECs were labeled by *Myo1A*^{ts}*>GFP*. **f,** Representative images of the *upd3-LacZ* staining in control (*attp2*) and *CncC* knockdown (*Myo1A*^{ts}*>CncC RNAi*) posterior midgut. **g,** Upper, schematic of the H2B-RFP EC labeling and tracing in control (*Mex*^{ts}*>UAS-H2B-RFP/attp2*) and *CncC* knockdown (*Mex*^{ts}*>UAS-H2B-RFP/UAS-CncC RNAi*) flies, flies were transferred to 30°C for 6 days at 4d to knock down *CncC* in ECs and labelled ECs. Lower, representative images of the RFP tracing pattern in midguts of 10d, 15d and 20d flies. **h,** Statistics of the ratio of RFP^{+} ECs to total ECs in selected areas of control and *CncC* knock down flies during aging. n, numbers of regions. attp2: n=22 (10d), 21 (15d), 24 (20d); *UAS-CncC RNAi*: n=22 (10d), 25 (15d), 24 (20d). **i,** Representative images of DHE staining of live midgut of 21d UT flies and 21d flies treated with NAC (DHE, red; GFP indicated ISCs/EBs). **j,** Statistics of PH3^{+} mitotic ISCs per midgut in UT- and NAC-treated flies at 23 days. n, numbers of midguts. n=20 (UT); n=19 (NAC). Data are mean ± SEM. Significance was determined using two-tailed unpaired *t* test. Scale bars, 10 μm (**c, e, f**), 20 μm (**b, g, i**). Source data are provided as a Source Data file.

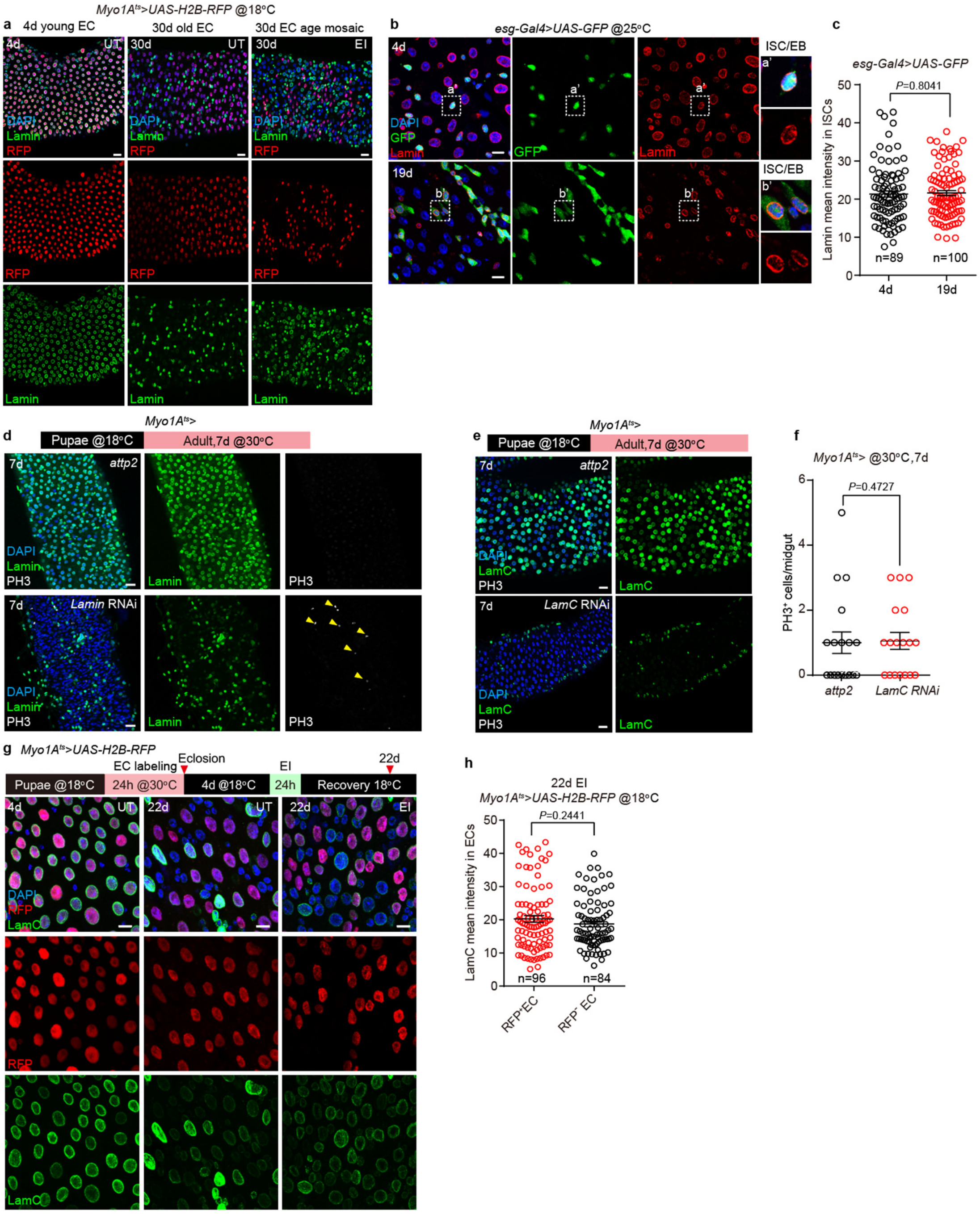

Supplementary Fig. 6 | Age-related degeneration of the Lamin in ECs

a, Representative images of Lamin staining in puparium-generated ECs (RFP^+) and newly generated ECs (RFP^-) in the UT and EI (bleomycin-fed 24h at 4d) midgut during aging. **b,** Representative images of Lamin staining in 4d and 19d *esg>UAS-GFP* midguts. ISCs and EBs are labeled with *esg>UAS-GFP*. **a'** and **b'** show higher magnification of Lamin staining on the ISC/EB nuclear membrane. **c,** Quantification of the Lamin fluorescence intensity in the ISC/EB of 4d and 19d UT midgut. n, numbers of ISC/EB. **d,** Upper, schematic of genetic manipulations. Lower, representative images of Lamin and PH3 staining in control (*attp2*) and *Myo1A^{ts}>Lamin RNAi* posterior midgut. Due to the knockdown of Lamin in polyploid ECs, the remaining Lamin staining is present in the diploid cells. Yellow arrowheads indicate the location of $PH3^+$ cells. **e,** Upper, schematic of genetic manipulations. Lower, representative images of LamC staining in control (*attp2*) and *Myo1A^{ts}>LamC RNAi* posterior midgut. Due to the knockdown of LamC in polyploid ECs, the remaining LamC staining is present in the diploid cells. **f,** Statistics of $PH3^+$ cells in *Myo1A^{ts}>LamC RNAi* midgut. n,

numbers of midguts. n=18 (*attp2*), 18 (*LamC RNAi*). **g,** Upper, the schematic shows the method for tracing the LamC staining in the midgut with puparium H2B-RFP labeled ECs. Lower, representative images of LamC staining in 4d and 22d UT and EI (bleomycin-fed) midguts. **h,** Statistics of LamC staining intensity in RFP$^+$ (old) ECs or RFP$^-$ (new) ECs in 22d EI midguts. n, numbers of ECs. Data are mean ± SEM. Significance was determined using two-tailed unpaired *t* test. Scale bars, 10 μm (**b, g**), 20 μm (**a, d, e**). Source data are provided as a Source Data file.

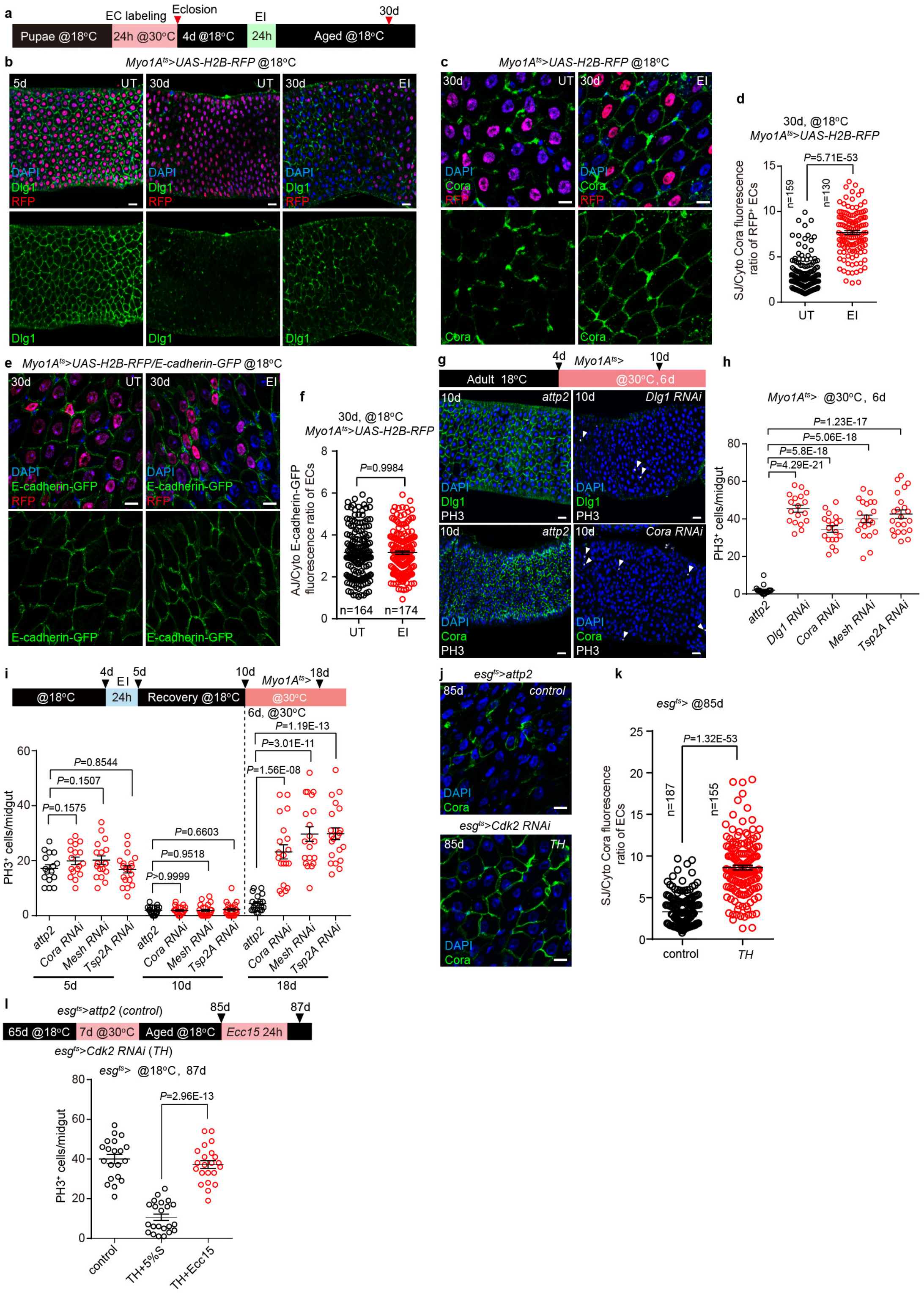

Supplementary Fig. 7 | Aged midgut septate junctions improved by age mosaic

a, The schematic shows the method for monitoring SJs in the midgut with puparium H2B-RFP labeled ECs. **b,** Representative images of the Dlg1 staining in UT and EI (bleomycin-fed 24h at 4d) midgut during aging. **c, d,** Representative images **c** and statistics **d** of the Cora staining in UT and EI (bleomycin-fed 24h at 4d) midgut during aging. **e, f,** Representative images **e** and statistics **f** of the E-cadherin staining in UT and EI (bleomycin-fed 24h at 4d) midgut during aging. **g,** Upper, schematic of the genetic manipulations. Lower, representative images of Dlg1/Cora and PH3 staining in control (*attp2*) and *Myo1A^{ts}>Dlg1/Cora RNAi* posterior midgut. White arrowheads indicate the locations of PH3^{+} cells. **h,** Statistics of PH3^{+} cells in midgut of control (*attp2*) and knockdown indicated SJ components in ECs. n, numbers of midguts. n (from left to right) =17, 18, 19, 22, 22. **i,** Upper, schematic of the method that knocked down SJs after the recovery from EI (bleomycin-fed). Lower, statistics of PH3^{+} cells in midguts at indicated days. n, numbers of midguts. n (from left to right) =16, 17, 18, 19, 19, 19, 23, 22, 20, 20, 20, 22. **j, k,** After the temporarily halting (TH) the ISC division in 65d hyperplastic midgut by *esg^{ts}>Cdk2 RNAi* for 7 days, representative images **j** and statistics **k** of the Cora staining in control and TH midgut at 85d. **l,** Upper, schematic of the design to test whether the oral infection of *Ecc15* could activate ISC proliferation after the TH. Lower, statistics the PH3^{+} cells in control (*esg^{ts}>attp2*) and TH (*esg^{ts}>Cdk2 RNAi*) midguts after *Ecc15* oral infection. n, numbers of midguts. n=20 (control), n=22 (TH+5%), n=22 (TH+*Ecc15*). Data are mean ± SEM. Significance was determined using two-tailed unpaired *t* test. n, number of measured ECs (**d, f, k**). Scale bars, 10 μm (**c, e, j**), 20 μm (**b, g**). Source data are provided as a Source Data file.

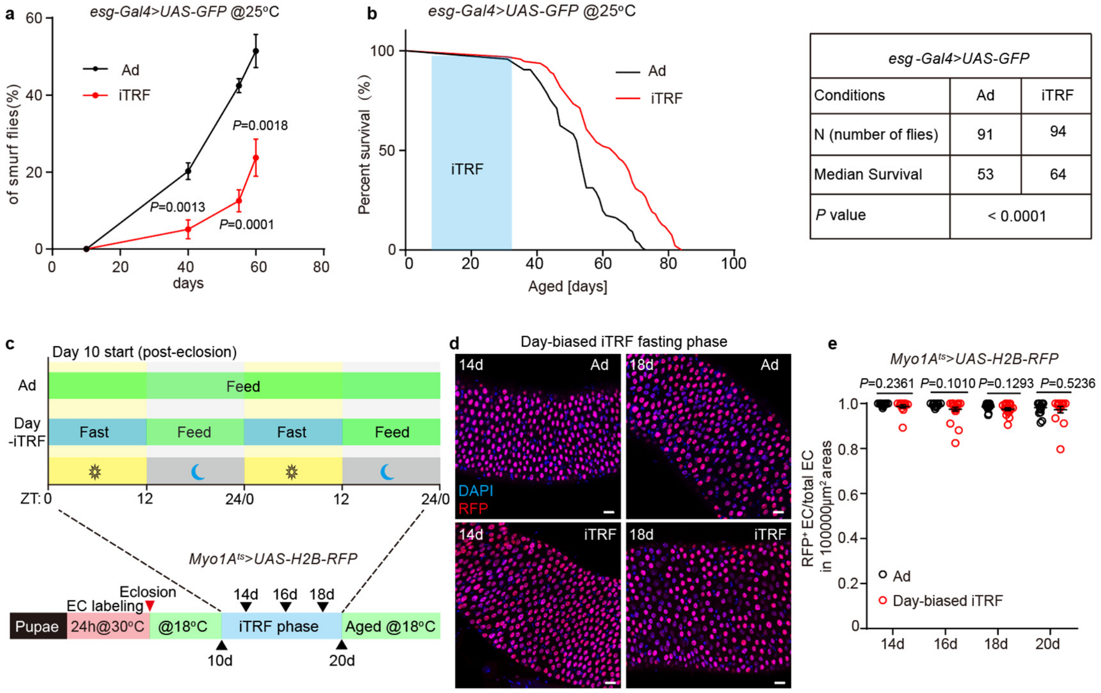

Supplementary Fig. 8 | iTRF improves midgut barrier function and extends lifespan

a, Smurf proportions of Ad and iTRF flies in a time course. n, numbers of flies. Ad: n=90 (10d), 84 (40d), 62 (55d), 36 (60d). iTRF: n=90 (10d), 81 (40d), 77 (55d), 49 (60d). **b,** Survival analysis of the Ad and iTRF flies. The light blue indicates the time window in which the iTRF fasting is in place. All flies are mated female flies. **c,** Schematic of day-biased iTRF treatment of puparium H2B-RFP EC-labeled flies. **d,** Representative images of the tracing pattern of puparium RFP-labeled ECs in 14d and 18d Ad and day-biased iTRF flies. **e,** Statistics of the ratio of RFP-labeled ECs to total ECs in midgut after Ad and day-biased iTRF treatment. n, numbers of regions. Ad: n=16 (14d), 14 (16d),18 (18d), 18 (20d); Day-biased iTRF: n=15 (14d), 24 (16d),17 (18d), 15 (20d). Data are mean ± SEM. Significance was determined using two-tailed unpaired *t* test. n, numbers of ECs. Scale bars, 20 μm. Source data are provided as a Source Data file.

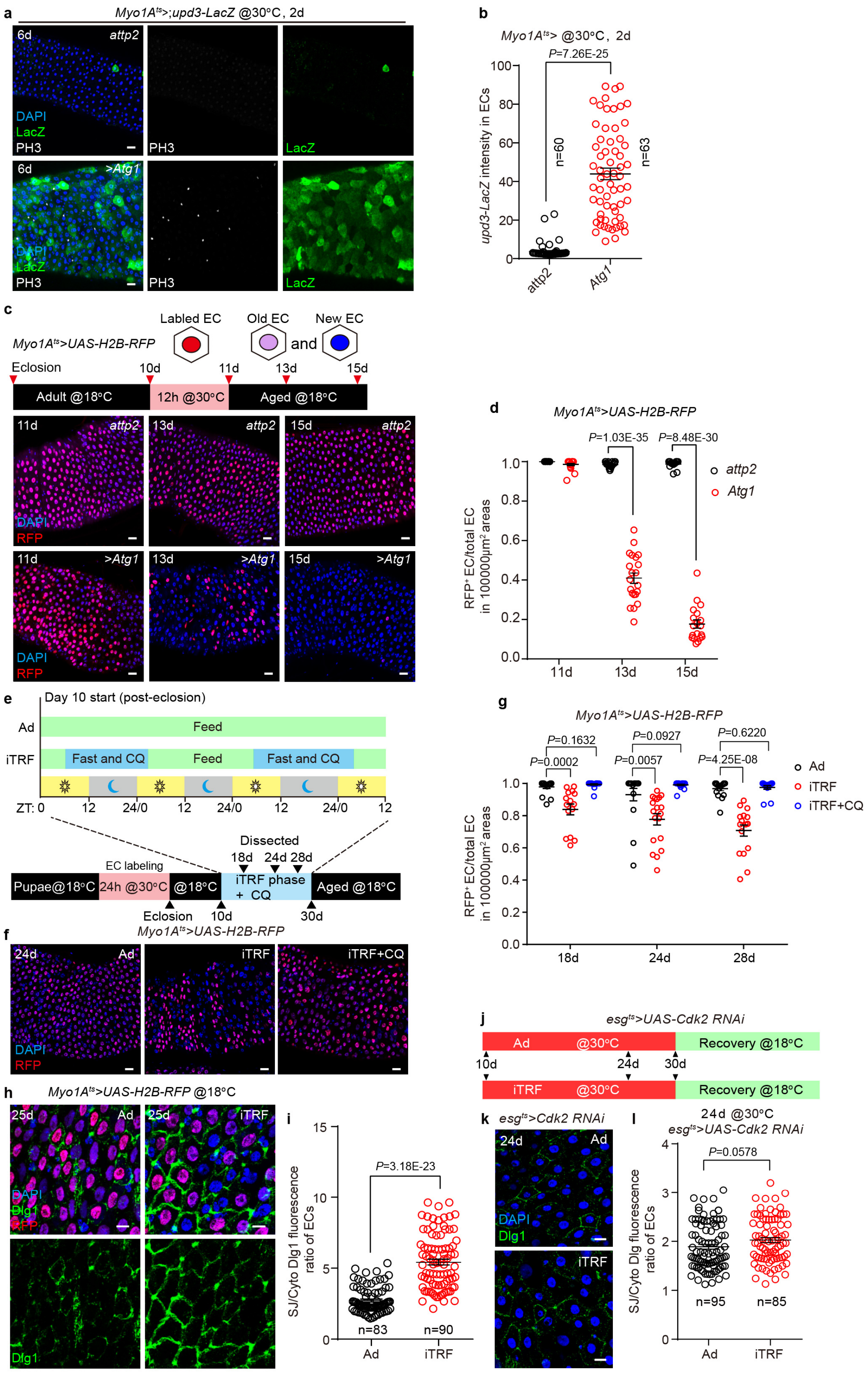
a
Myo1A^{ts}>;upd3-LacZ @30°C, 2d
6d attp2
DAPI
LacZ
PH3
PH3
LacZ
6d >Atg1
DAPI
LacZ
PH3
PH3
LacZ
b
Myo1A^{ts}> @30°C, 2d
P=7.26E-25
upd3-LacZ intensity in ECs
n=60
n=63
attp2
Atg1
c
Myo1A^{ts}>UAS-H2B-RFP
Labled EC
Old EC
and
New EC
Eclosion
10d
11d
13d
15d
Adult @18°C
12h @30°C
Aged @18°C
11d attp2
13d attp2
15d attp2
DAPI
RFP
11d >Atg1
13d >Atg1
15d >Atg1
DAPI
RFP
d
Myo1A^{ts}>UAS-H2B-RFP
P=1.03E-35
P=8.48E-30
attp2
Atg1
RFP^{+} EC/total EC in 100000μm^{2} areas
11d
13d
15d
e
Day 10 start (post-eclosion)
Ad
Feed
iTRF
Fast and CQ
Feed
Fast and CQ
ZT: 0
12
24/0
12
24/0
12
24/0
12
Dissected
18d
24d
28d
EC labeling
Pupae@18°C
24h @30°C
@18°C
iTRF phase + CQ
Aged @18°C
Eclosion
10d
30d
f
Myo1A^{ts}>UAS-H2B-RFP
24d Ad
iTRF
iTRF+CQ
DAPI
RFP
g
Myo1A^{ts}>UAS-H2B-RFP
P=0.1632
P=0.0927
P=0.6220
P=0.0002
P=0.0057
P=4.25E-08
Ad
iTRF
iTRF+CQ
RFP^{+} EC/total EC in 100000μm^{2} areas
18d
24d
28d
h
Myo1A^{ts}>UAS-H2B-RFP @18°C
25d Ad
25d iTRF
DAPI
Dlg1
RFP
Dlg1
i
P=3.18E-23
SJ/Cyto Dlg1 fluorescence ratio of ECs
n=83
n=90
Ad
iTRF
j
esg^{ts}>UAS-Cdk2 RNAi
Ad
@30°C
Recovery @18°C
10d
24d
30d
iTRF
@30°C
Recovery @18°C
k
esg^{ts}>Cdk2 RNAi
24d Ad
DAPI
Dlg1
iTRF
l
24d @30°C
esg^{ts}>UAS-Cdk2 RNAi
P=0.0578
SJ/Cyto Dlg fluorescence ratio of ECs
n=95
n=85
Ad
iTRF

Supplementary Fig. 9 | Autophagy is required for iTRF- induced EC turnover

a, b, Representative images **a** and statistics **b** of the *upd3-LacZ* staining in control (*attp2*) and EC Atg1 overexpression (*Myo1A^{ts}>UAS-Atg1*) midgut. PH3 staining shows ISC overproliferation in *Myo1A^{ts}>UAS-Atg1* midgut. **c,** Upper, schematic of the tracking of puparium H2B-RFP labeled ECs when Atg1 is overexpressed for 12d in ECs on an Ad diet. Lower, representative images of H2B-RFP tracing pattern of ECs in control (*attp2*) and EC Atg1 overexpression (*Myo1A^{ts}>UAS-Atg1*) midgut at 11d, 13d, 15d. **d,** Statistics of the ratio of RFP-labeled ECs to total ECs in control (*attp2*) and EC Atg1 overexpression (*Myo1A^{ts}>UAS-Atg1*) midgut at 11d, 13d, 15d. n, numbers of regions. attp2: n=17 (11d), 20 (13d), 18 (15d); Atg1: n=19 (11d), 21 (13d), 19 (15d). **e,** Schematic of CQ (Chloroquine) feeding during the iTFR fasting phase (iTRF+CQ). **f,** Representative images of the puparium labeled H2B-RFP tracking pattern in 24d iTRF and iTRF+CQ posterior midgut. **g,** Statistics of the ratio of RFP-labeled ECs to total ECs in 18d, 24d, and 28d iTRF and iTRF+CQ posterior midgut. n, numbers of regions. Ad: n=17 (18d), 16 (24d), 17 (28d); iTRF: n=15 (18d), 19 (24d), 17 (28d); iTRF+CQ: n=19 (18d), 19 (24d), 15 (28d). **h, i,** Representative images **h** and statistics **i** of Dlg1 staining in Ad and iTRF posterior midgut with puparium H2B-RFP labeled ECs. **j,** Schematic of the prevention of age mosaic formation by blocking ISC divisions (*esg^{ts}>Cdk2 RNAi*) in Ad and iTRF flies. **k, l,** Representative images **k** and statistics **l** of Dlg1 staining in 24d Ad and iTRF posterior midgut with age mosaic prevention (*Cdk2 RNAi*). Data are mean ± SEM. Significance was determined using two-tailed unpaired *t* test. n, numbers of ECs (**b, i, l**). Scale bars, 20 μm (**a**, **c**, **f**), 10 μm (**h**, **k**). Source data are provided as a Source Data file.
